# Magnitude and factors associated with low birth weight among newborns in public health facilities of Mekelle City, northern Ethiopia: a multi-center study

**DOI:** 10.3389/fped.2024.1455248

**Published:** 2024-12-24

**Authors:** Gebremichael Aregawi Teklehaimanot, Kahsay Zenebe Gebreslasie, Woldu Mammo Werid, Berhanu Gebresilassie, Gebregziabher Kidanemariam, Etsay Weldekidan Tsegay, Zenawi Hagos Gufue, Meresa Berwo Mengesha

**Affiliations:** ^1^ Department of Midwifery, College of Medicine and Health Sciences, Adigrat University, Adigrat, Ethiopia; ^2^ Department of Midwifery, College of Health Sciences, Mekelle University, Mekelle, Ethiopia; ^3^ Department of Midwifery, College of Medicine and Health Sciences, Axum University, Axum, Ethiopia; ^4^ Department of Modern and Traditional Medicine Research, Tigray Health Research Institute, Mekelle, Ethiopia; ^5^ Department of Public Health, College of Medicine and Health Sciences, Adigrat University, Adigrat, Ethiopia

**Keywords:** low birth weight, maternal characteristics, newborn characteristics, Mekelle, northern Ethiopia

## Abstract

**Background:**

Low birth weight is a key determinant of child survival, significantly influencing rates of infant and childhood mortality, morbidity, and disability. While some studies have been conducted in our region, there is still a gap in evidence regarding the maternal characteristics associated with low birth weight. Hence, this study aimed to determine the proportion of newborns with low birth weight and determinant factors, particularly focusing on maternal characteristics.

**Method:**

A facility-based cross-sectional study was conducted from 21 March to 20 April 2020 involving mothers and their newborns at selected public health facilities in Mekelle City. The sample included 447 participants, with two public hospitals and three health centers chosen by a lottery method. Systematic random sampling was applied to select mother–newborn pairs. Data were collected using a structured, interviewer-administered questionnaire and analyzed using the Statistical Package for Social Sciences version 21. Bivariate and multivariate logistic regression analyses, with a 95% confidence interval (CI), were used to identify factors associated with low birth weight.

**Results:**

The study included 447 mothers of newborns, achieving a 100% response rate. The proportion of low birth weight was 14.3%. Significant factors associated with low birth weight included attending the first antenatal care (ANC) visit in the third trimester [adjusted odds ratio (AOR) = 3.66, 95% CI: 1.28–10.44], not receiving additional nutrition during pregnancy (AOR = 4.16, 95% CI: 1.38–12.58), experiencing obstetric complications during the current pregnancy (AOR = 7.72, 95% CI: 2.76–21.59), and a gestational age at birth of less than 37 weeks (AOR = 5.36, 95% CI: 1.96–14.67).

**Conclusion and recommendation:**

This study revealed a substantial incidence of low birth weight. The initiation of the first antenatal care visit in the third trimester, failure to supplement nutrition during pregnancy, the occurrence of obstetric complications during pregnancy, and a gestational age at birth less than 37 weeks were all found to be significantly correlated with this condition. It is recommended that policymakers strengthen maternal and child health services, especially through the focused ANC program, to improve outcomes. Health facilities should promote awareness of the importance of initiating ANC visits early, with an emphasis on nutritional counseling throughout pregnancy.

## 1 Introduction

Birth weight, defined as the initial weight of a newborn or fetus at birth, is ideally measured within the first hour of life to avoid inaccuracies due to early postnatal weight loss (1). Accurate recording of birth weight is essential, as it provides critical information for assessing the health and survival prospects of the newborn (2).

According to the World Health Organization (WHO), low birth weight (LBW) is classified as a birth weight of less than 2,500 g (5.5 pounds) (3). This classification is based on epidemiological evidence indicating that infants with a birth weight below 2,500 g have approximately 20 times the risk of mortality compared to those with higher birth weight (4). Low birth weight is more common in developing countries and is associated with a range of adverse health outcomes (5).

Low birth weight can result from preterm birth (delivery before 37 completed weeks of gestation) or from being small for gestational age (SGA), which is defined as a birth weight below the 10th percentile for gestational age (GA) (6). In some cases, infants born at term may also have low birth weight without meeting the criteria for SGA (7).

Globally, over 20 million infants—about 15.5% of all births—are born with low birth weight (8). In developing countries, the rate of LBW is significantly higher at 16.5% compared to just 7% in developed regions (9). Approximately 95% of LBW infants are born in developing countries, with the highest incidence in South-Central Asia (27%), followed by sub-Saharan Africa, which accounts for 13%–15% of global LBW cases (5). In Ethiopia, 24% of all children are underweight (below −2 SD), and 7% are severely underweight (below −3 SD), with higher prevalence in rural areas compared to urban ones (25% vs. 13%) (10). Low birth weight is a pressing public health issue globally, as it not only predicts perinatal mortality and morbidity but also heightens the risk of non-communicable diseases, such as diabetes and cardiovascular disease, later in life (11). In Ethiopia, the neonatal mortality rate stands at 29 deaths per 1,000 live births, with LBW being a major contributor (10).

Research highlights that LBW is influenced by numerous maternal socio-demographic and obstetric factors, including maternal age, educational level, marital status, parity, antenatal care (ANC) follow-up, pregnancy complications, and gestational age in addition to the lack of organizational and structural interventions (12–14).

Despite existing studies, there is a gap in evidence on maternal characteristics related to low birth weight in Ethiopia. Furthermore, recent data on the prevalence and risk factors of LBW in specific regions are sparse. Since LBW is a critical health indicator, understanding its prevalence and associated factors across various areas is essential for public health planning and interventions.

Despite improvements in maternal and child health services in developing countries, including Ethiopia, high rates of low birth weight and neonatal mortality persist. Despite initiatives such as strengthening primary healthcare and community interventions in Ethiopia, morbidity and mortality from low birth weight and prematurity remain high due to the lack of structural and organizational continuum of care interventions (15).

To the best of our knowledge, no previous studies have addressed this topic within the specific study area. Therefore, this research seeks to provide a foundation for stakeholders, health professionals, regional health bureaus, and health institutions, informing their planning efforts. This study aimed to address the issue of LBW by identifying the maternal and newborn characteristics linked to it in Mekelle City, Tigray, northern Ethiopia.

## 2 Methods and materials

### 2.1 Study area and period

The study was conducted at public health institutions in Mekelle City, the capital of Tigray Regional State. Administratively, the city is divided into seven sub-cities and is located in the southeastern zone of the Tigray region, approximately 781 km north of Addis Ababa, the capital of Ethiopia. Mekelle has 1 referral hospital, 2 general hospitals, and 10 health centers. This study focused on institutions within Mekelle City that provide 24-h delivery services.

### 2.2 Study design

An institution-based cross-sectional study was conducted from 21 March to 20 April 2020.

### 2.3 Study population

The source population included all mothers with newborns who gave birth in public health institutions in Mekelle City, northern Ethiopia. The study population consisted of selected mothers with newborns who delivered at selected public health facilities in Mekelle City during the data collection period.

### 2.4 Inclusion and exclusion criteria

All newborns and their mothers who delivered in selected public health institutions in Mekelle City during the study period were included; however, multiple births and cases of congenital anomalies were excluded from the beginning.

### 2.5 Sample size and sampling procedure

The sample size was determined using a single-population proportion formula with the following assumptions: a previous prevalence of LBW of 24.4% (16), a 5% margin of error, a 95% confidence interval (CI) (*α* = 0.05), and a non-response rate of 5%.

After accounting for the 5% non-response rate, the initial sample size was 298. Since a two-stage sampling technique was employed, a design effect of 1.5 was applied, yielding a final sample size of 447 (298 × 1.5). Mekelle has 3 public hospitals and 10 health centers that offer delivery services. Two public hospitals (Ayder Specialized Referral Hospital and Mekelle Hospital) and three health centers (Mekelle Health Center, Adiha Health Center, and Semen Health Center) were selected using the lottery method.

**Figure 1:**
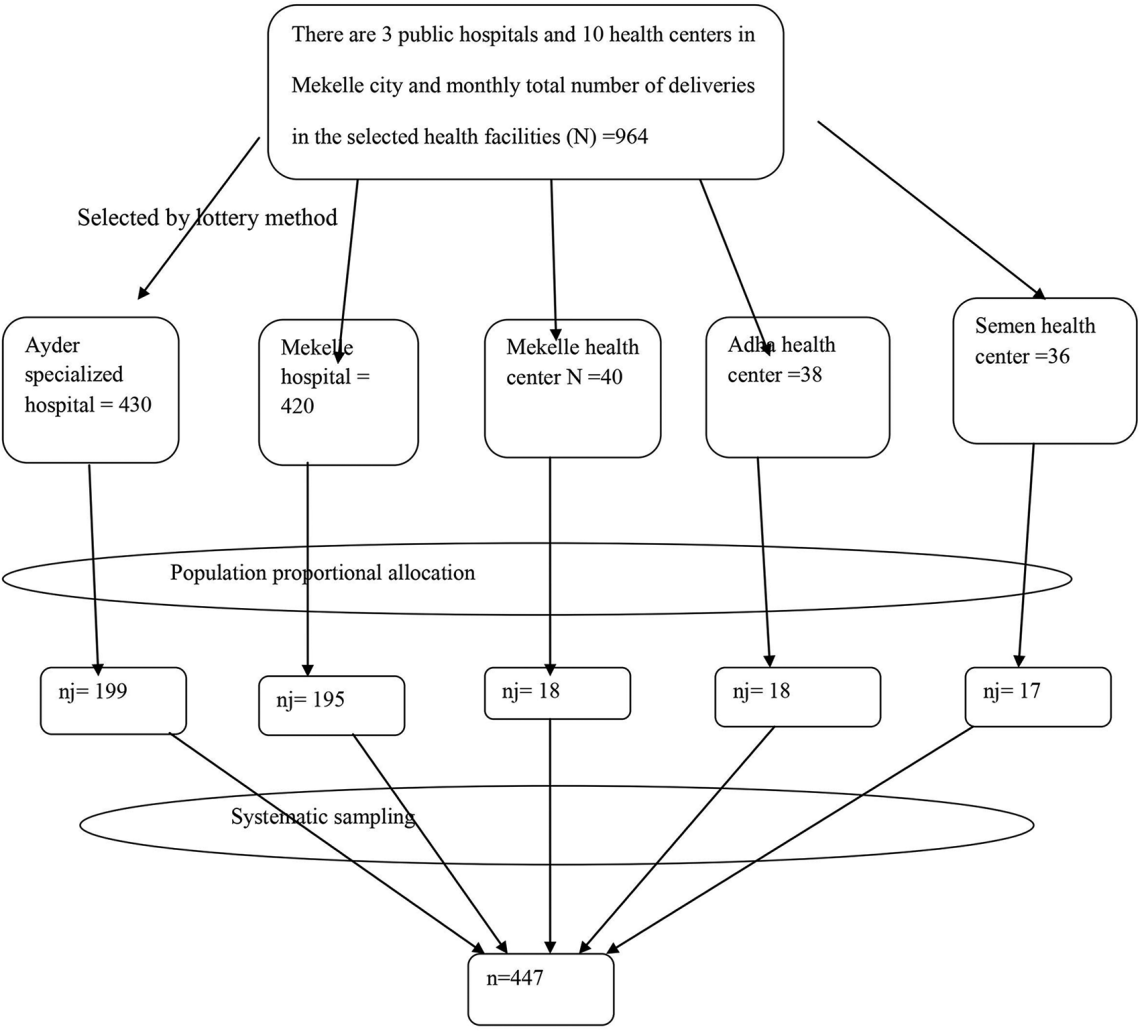
Schematic presentation of the sampling procedure for the study conducted on LBW newborns in Mekelle City, 2020.

The sample was allocated proportionally to each health facility based on the population size of mothers delivering in public health institutions, as indicated by monthly delivery reports prior to data collection. After assigning the total sample size to each health facility proportionally, systematic sampling was used to select mother–newborn pairs. The sampling interval (*K*) was calculated by dividing the total expected monthly deliveries (964, *N*) by the sample size of 447 (*n*) at each data collection site, resulting in *K* = 2. Every second eligible client was included until the required sample size was reached (Figure 1).

The *K*th interval for each health facility was calculated as follows: Ayder Specialized Referral Hospital, with a total monthly delivery estimate of 430 (*N*) and a sample size of 199 (*n*), resulted in a sampling interval of *K* = 2; Mekelle Hospital, with 420 monthly deliveries (*N*) and a sample size of 195 (*n*), also had a sampling interval of *K* = 2; Mekelle Health Center, with 40 monthly deliveries (*N*) and a sample size of 18 (*n*), had a sampling interval of *K* = 2; Adha Health Center, with 38 monthly deliveries (*N*) and a sample size of 18 (*n*), had a sampling interval of *K* = 2; and Semen Health Center, with 36 monthly deliveries (*N*) and a sample size of 17 (*n*), had a sampling interval of *K* = 2.

### 2.6 Data collection procedure

Data were collected through face-to-face interviews and by reviewing medical charts of ANC records for study participants using structured questionnaires adapted from various relevant sources. Mothers were asked about their socio-demographic and maternal factors within 24 h of delivery. Gestational age was estimated based on the number of days between the first day of the last menstrual period (LMP) and the date of birth, expressed in completed weeks, or by early ultrasound examination conducted in the first trimester. Birth weight was measured within 1 h of delivery using a standard beam balance and a scale with an accuracy of 100 g. The scale was checked and calibrated before weighing. The principal investigator checked the scales daily, and data collectors verified and adjusted them to zero between measurements.

Data collection was performed by five BSc Midwifery team members and supervised by two experienced individuals with BSc degrees in midwifery.

### 2.7 Variables of the study

#### 2.7.1 Dependent variable

 • Birth weight

#### 2.7.2 Independent variables

 • Socio-demographic factors: age, religion, marital status, educational level, family income, occupation, and residence.
 • Obstetric factors: parity, gestational age, mode of delivery, and obstetric complications.
 • Service-related factors: ANC visits, iron with folic acid taken, tetanus toxoid (TT) vaccine, use of insecticide-treated net (ITN), and hemoglobin level.
 • Medical factors: malaria, anemia, and HIV.
 • Nutritional factors: nutritional counseling and taking extra meals during pregnancy.

### 2.8 Operational definitions

 • Low birth weight: newborns weighed less than 2500 g.
 • Premature (preterm): infants born before 37 completed weeks of gestation and after 28 weeks of gestation (28–36+^6^ weeks).
 • Obstetric complication during pregnancy: if mothers have one of the following obstetric complications [hypertensive disorders of pregnancy, *antepartum* hemorrhage (APH), premature rupture of membrane (PROM), and oligohydramnios].

### 2.9 Data quality assurance and management

The validity of the questionnaire was ensured by adapting it from relevant studies conducted by other researchers and modifying it to fit the local context. The questionnaire was pre-tested on 5% of the total sample size at Quaha Health Center, located 10 km from Mekelle City, and adjustments were made prior to the actual data collection.

Training was provided to data collectors and supervisors on calculating gestational age, measuring newborn weight, approaching study participants, and obtaining their consent for interviews. Data collection was conducted by five BSc midwives fluent in Tigrigna and supervised by two BSc midwives. Before each interview, data collectors explained the purpose and significance of the study to participants, who were then invited to take part in the study.

The questionnaire was originally prepared in English, then translated into the local language (Tigrigna), and finally back-translated into English to ensure accuracy in meaning.

### 2.10 Data processing and analysis

The questionnaire was checked for completeness and coded prior to data entry. The data were then cleaned and entered into EpiData version 4.2 and subsequently exported to Statistical Package for Social Sciences (SPSS) version 21 for analysis. Bivariate analysis was conducted to examine the association between independent and dependent variables. Variables showing statistically significant results (*p* < 0.2 in binary logistic regression) were included in a multivariable logistic regression analysis to identify predictive factors for LBW while controlling for confounding variables. Variables with *p* <0.05 in multivariable logistic regression were considered predictive factors associated with LBW. Odds ratios with 95% confidence intervals were used to measure the strength of association between independent and dependent variables. Finally, results are presented in tables, figures, and text.

## 3 Results

### 3.1 Socio-demographic characteristics of participants

The study included 447 mothers and their newborns, achieving a 100% response rate. Participants’ ages ranged from 17 to 48 years, with a mean age of 27 years (SD ±5.6 years). The majority of participants, 344 (77%), were in the 20–34 age group. Among the 447 participants, 419 (93.7%) were married, while 298 (66.7%) were housewives. Most participants, 410 (91.7%), identified as Orthodox Christians. Regarding education, 169 participants (37.8%) had completed secondary school. The results showed that 387 participants (86.6%) resided in urban areas, and 327 (73.2%) reported a monthly income level of more than 2,000 ETB (Table 1).

**Table 1:** Socio-demographic characteristics of study participants in public health facilities of Mekelle City, northern Ethiopia, 2020 (*N* = 447).

Variables	Categories	Frequency	Percentage
Age of participants	Less than 20 years old	46	10.3
	20–34 years old	344	77.0
	Greater than 35 years old	57	12.7
Marital status of participants	Unmarried	26	5.8
	Married	419	93.7
	Divorced	2	0.5
Occupation of participants	Housewife	298	66.7
	Government employee	73	16.3
	Non-government employee	7	1.6
	Merchant	63	14.1
	Others	6	1.3
Religion of participants	Orthodox	410	91.7
	Muslim	37	8.3
Educational level of participants	Unable to read and write	13	2.9
	Able to read and write	24	5.4
	Primary school (1–8)	120	26.8
	Secondary school (9–12)	169	37.8
	College and above	121	27.1
Residency of participants	Rural	60	13.4
	Urban	387	86.6
Monthly income of participants (ETB)	<500	23	5.1
	500–1,000	24	5.4
	1,001–2,000	73	16.3
	>2,000	327	73.2

### 3.2 Proportion of low birth weight

**Figure 2:**
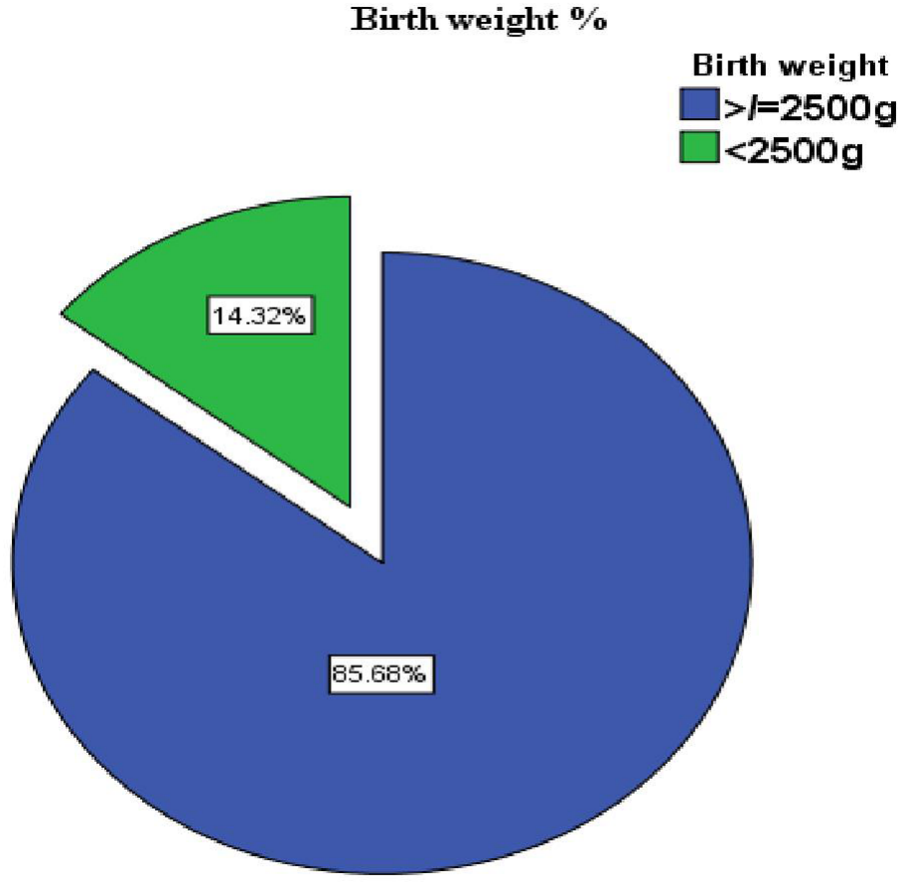
Prevalence of low birth weight in public health facilities of Mekelle City, Ethiopia, 2020.

In this study, the proportion of LBW was 14.3%. Newborn birth weights ranged from 1,500 to 4,500 g, with a mean birth weight of 3,047 g (SD ±536 g) (Figure 2).

### 3.3 Antenatal care characteristics

Nearly all participants (443, 99.1%) had attended ANC visits, with 346 (78.1%) having fewer than four visits and 79 (17.8%) starting ANC visits in the third trimester. The majority of participants (394, 88.9%) received iron and folic acid supplementation during their current pregnancy. Hemoglobin levels were measured in 444 participants (99.3%) during ANC follow-up or before delivery, with a minimum recorded hemoglobin level of 7.9 g/dl; among the participants, 34 participants (7.7%) were diagnosed with anemia. Most participants (399, 90.1%) received counseling on danger signs during their current pregnancy. In addition, 400 participants (90.3%) reported receiving dietary counseling, and 359 (81%) were consuming extra meals. The majority (427, 98.6%) had negative HIV test results, and 405 (97.6%) were non-reactive on The venereal disease research laboratory (VDRL) testing. Furthermore, 389 participants (87.8%) had received a tetanus toxoid vaccination during or before their current pregnancy (Table 2).

**Table 2:** Maternal-related characteristics of study participants of LBW in public health facilities of Mekelle City, northern Ethiopia, 2020 (*N* = 447).

Variables	Categories	Frequency	Percentage
ANC follow-up during the current pregnancy	Yes	443	99.1
	No	4	0.9
Number of ANC visits	<4 ANC visits	346	78.1
	>/=4 ANC visits	97	21.9
Time of starting the ANC visit	First trimester	231	52.2
	Second trimester	133	30
	Third trimester	79	17.8
Have you taken iron with folic acid tablets during this pregnancy?	Yes	394	88.9
	No	49	11.1
Hemoglobin level	<11 g/dl	34	7.7
	>/=11 g/dl	410	92.3
Have you counseled danger signs during this pregnancy?	Yes	399	90.1
	No	44	9.9
Have you screened for HIV during this pregnancy?	Yes	433	97.7
	No	10	2.3
If yes, what was your HIV result?	NR	427	98.6
	R	5	1.2
	Unknown	1	0.2
Have you got nutritional counseling during this pregnancy?	Yes	400	90.3
	No	43	9.7
Have you taken additional nutrition than usual?	Yes	359	81.0
	No	84	19.0
Have you taken TT immunization?	Yes	389	87.8
	No	54	12.2
If yes, how many times did you take TT immunization?	≤4	306	78.5
	5	83	21.5
Have you screened VDRL during the current pregnancy?	Yes	415	93.7
	No	28	6.3
What was the result of VDRL during this pregnancy?	NR	405	97.6
	R	3	0.7
	Unknown	7	1.7

NR, non-reactive; R, reactive; TT, tetanus toxoid.

### 3.4 Maternal and newborn-related factors

Two hundred twenty-one participants (49.4%) had given birth to two to four children. A total of 396 (88.6%) and 442 (98.9%) participants reported that their current pregnancies were planned and wanted, respectively. Seventy-four participants (16.6%) experienced obstetric complications during the current pregnancy, with 36 (48.6%) having a hypertensive disorder.

During the current pregnancy, 217 participants (48.5%) used ITNs. Forty-three participants (9.6%) had a history of abortion, 13 (2.9%) had a history of low birth weight, 11 (2.5%) had a history of preterm delivery, and 9 (2%) had a history of stillbirth.

**Figure 3:**
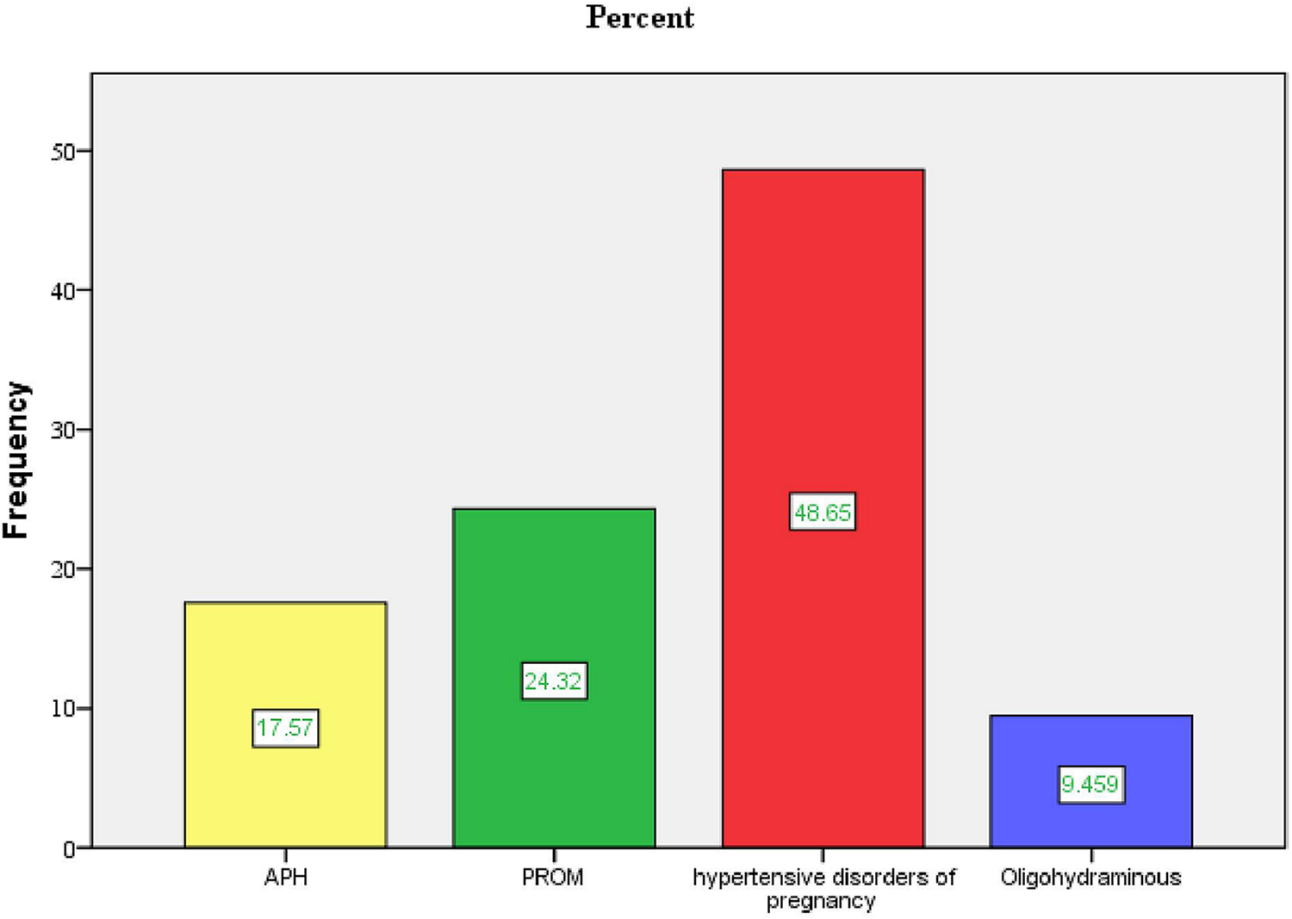
Type of obstetrical complications during current pregnancy in public health facilities of Mekelle City, northern Ethiopia, 2020.

**Figure 4:**
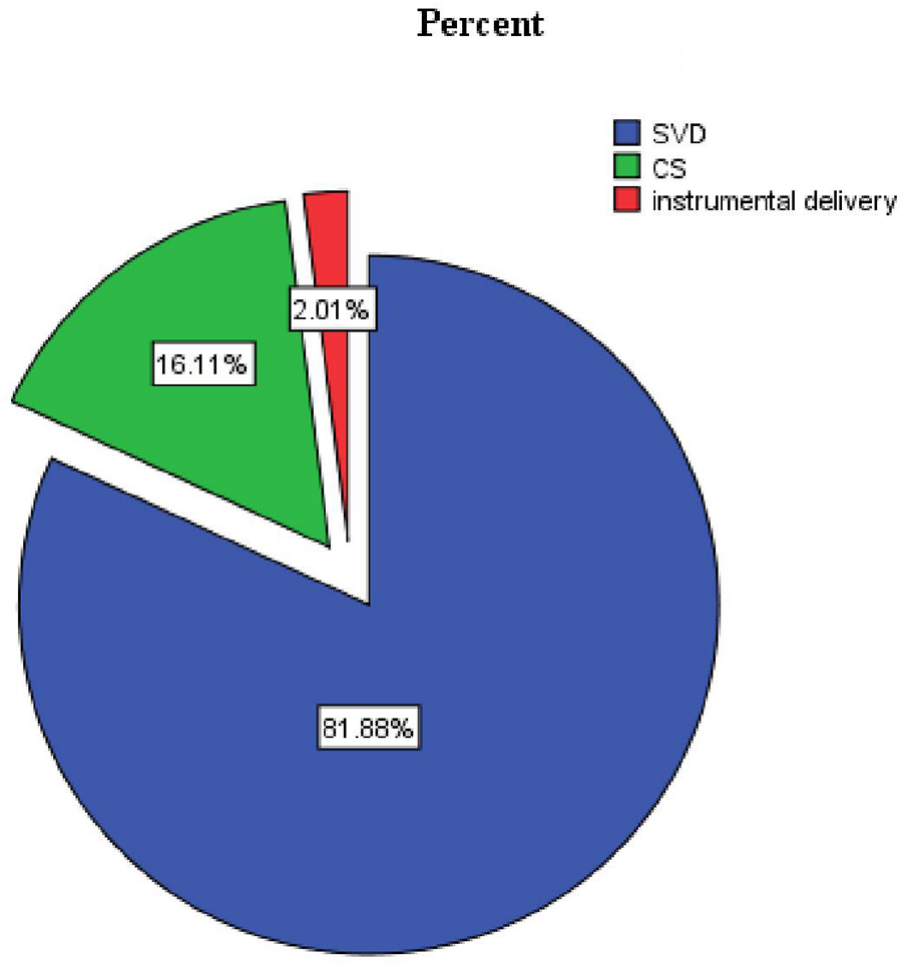
Proportion of the mode of delivery of LBW in public health facilities of Mekelle City, Ethiopia, 2020.

**Figure 5:**
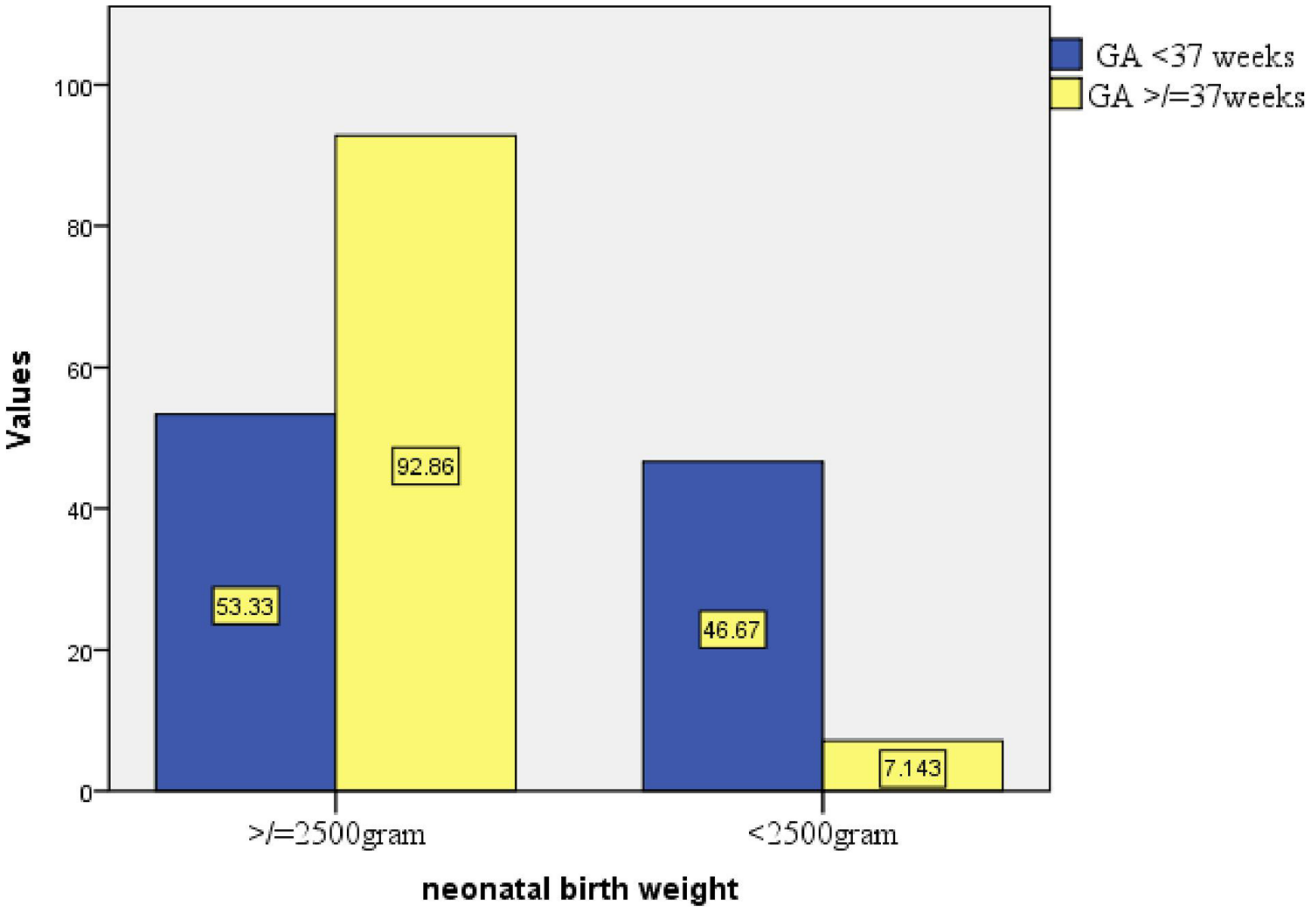
Proportion of birth weight based on GA at birth in public health facilities of Mekelle City, northern Ethiopia, 2020.

Three hundred sixty-six participants (81.9%) delivered via spontaneous vaginal delivery, and 322 (84.3%) had a gestational age of ≥37 weeks at the time of delivery (Table 3 and Figures 3–5).

**Table 3:** Maternal-related characteristics of study participants of LBW in public health facilities of Mekelle City, northern Ethiopia, 2020 (*N* = 447).

Variables	Categories	Frequency	Percentage
Parity	1	169	37.8
	2–4	221	49.4
	≥5	57	12.8
Planned	Yes	396	88.6
	No	51	11.4
Wanted	Yes	442	98.9
	No	5	1.1
Obstetric complications during the current pregnancy	Yes	74	16.6
	No	373	83.4
Did you use ITN (bed net) during this pregnancy?	Yes	217	48.5
	No	230	51.5
Did you have a malaria attack during this pregnancy?	Yes	2	0.4
	No	445	99.6
History of abortion	Yes	43	9.6
	No	404	90.4
History of low birth weight	Yes	13	2.9
	No	434	97.1
History of preterm birth	Yes	11	2.5
	No	436	97.5
History of stillbirth	Yes	9	2.0
	No	438	98.0
Gestational age	<37 weeks	60	15.7
	>37 weeks	322	84.3

### 3.5 Factors associated with low birth weight

Multivariable logistic regression analysis identified several factors significantly associated with low birth weight. These factors included initiating the first ANC visit in the third trimester, lack of additional nutrition during the current pregnancy, the presence of obstetric complications, and a gestational age at birth of less than 37 weeks.

Mothers who initiated their first ANC visit in the third trimester were 3.66 times more likely to give birth to a LBW neonate compared to those who started ANC in the first trimester [adjusted odds ratio (AOR) = 3.66, 95% CI: 1.280–10.439].

Mothers who did not receive additional nutrition during the current pregnancy were 4.16 times more likely to deliver an LBW neonate than those who did (AOR = 4.16, 95% CI: 1.377–12.583).

Mothers who experienced obstetric complications during the current pregnancy had 7.72 times higher odds of delivering an LBW neonate compared to those without obstetric complications (AOR = 7.72, 95% CI: 2.762–21.594).

The odds of a neonate having low birth weight were 5.36 times higher for those born before 37 weeks of gestation compared to those born at or after 37 weeks (AOR = 5.36, 95% CI: 1.957–14.671) (Table 4).

**Table 4:** Bivariate and multivariable logistic regression analyses of factors associated with low birth weight in public health facilities of Mekelle City, 2020 (*N* = 447).

Variables	Low birth weight		COR (95% CI)	AOR (95% CI)	*p*-value
	Yes	No			
Age					
<20 years old	22 (47.8%)	24 (52.8%)	2.567 (1.124–5.863)	3.543 (0.477–26.328)	0.103
20–34 years old	27 (7.8%)	317 (92.2%)	0.238 (0.117–0.484)	0.657 (0.121–3.576)	
≥35 years old	15 (26.3%)	42 (73.3%)	1	1	
Monthly income (ETB)					
<500	11 (47.8%)	12 (52.2%)	10.612 (4.268–26.390)	1.461 (0.226–9.435)	0.176
500–1,000	11 (45.8%)	13 (54.2%)	9.796 (3.993–24.030)	1.856 (0.351–9.825)	
1,001–2,000	16 (21.9%)	57 (78.1%)	3.250 (1.640–6.441)	0.793 (0.237–2.659)	
>2,000	26 (8%)	301 (92%)	1	1	
Hemoglobin level (g/dl)					
<11	11 (32.4%)	23 (67.6%)	3.443 (1.583–7.489)	0.644 (0.113–3.668)	0.982
≥11	50 (12.2%)	360 (87.8%)	1	1	
Time of start of ANC visit					
First trimester	18 (7.8%)	213 (92.2%)	1	1	
Second trimester	15 (11.3%)	118 (88.7%)	1.504 (0.731–3.094)	0.987 (0.292–3.340)	
Third trimester	28 (35.4%)	51 (64.6%)	6.497 (3.337–12.649)	**3.656 (1.280–10.439)** **^a^**	**0.012**
Nutritional counseling					
Yes	42 (10.5%)	358 (89.5%)	1	1	
No	19 (44.2%)	24 (55.8%)	6.748 (3.414–13. 340)	1.526 (0.327–7.120)	0.897
Took additional nutrition					
Yes	22 (6.1%)	337 (93.9%)	1	1	
No	39 (46.4%)	45 (53.6%)	13.276 (7.227–24.389)	**4.162 (1.377–12.583)** **^a^**	**0.001**
Parity					
1	33 (19.5%)	136 (80.5%)	0.679 (0.337–1.370)	2.092 (0.336–13.018)	0.458
2–4	16 (7.2%)	205 (92.8%)	0.219 (0.100–0.476)	1.108 (0.185–6.631)	
≥5	15 (26.3%)	42 (73.7%)	1	1	
Planned					
Yes	36 (9.1%)	360 (90.9%)	1	1	
No	28 (54.9%)	23 (45.1%)	12.174 (6.360–23.303)	1.563 (0.409–5.971)	0.241
Gestational age at birth (weeks)					
<37	28 (46.7%)	32 (53.3%)	11.375 (5.872–22.033)	**5.358 (1.957–14.671)** **^a^**	**0.001**
≥37	23 (7.1%)	299 (92.9%)	1	1	
Obstetric complications during the current pregnancy					
Yes	36 (48.6%)	38 (51.4%)	11.673 (6.427–21.201)	**7.723 (2.762–21.594)** **^a^**	**0.000**
No	28 (7.5%)	345 (92.5%)	1	1	

COR, crude odds ratio.

*p*-value <0.2 for bivariate logistic regression. *p*-value <0.05 for multivariable logistic regression.

^a^ Significantly associated with multivariable logistic regression.

## 4 Discussion

This institutional-based cross-sectional study aimed to determine the magnitude of low birth weight and its predictive factors, revealing that 14.3% of the newborns had low birth weight. This finding is consistent with studies conducted in Kenya (12.3%), Sudan (13%) (17–21), Gondar (17.4%), and Suhul Shire (11.5%) (22–24).

The findings of this study are higher than those reported in Colombia (8.7%), Indonesia (10.2%), Gambia (10.5%), Ghana (8.7%), Wolaita Sodo, South Ethiopia (8.1%), Addis Ababa (8.8%), Tigray (10.5%), and Axum (8.8%) (17, 19, 23, 25–29). This difference may be attributed to geographical variations, disparities in health service utilization, the nutritional status of mothers during pregnancy, differences in the study area, sample size, and the inclusion of preterm births, which could have contributed to the higher prevalence of low birth weight in this study. However, the prevalence of LBW in this study is lower than those in studies conducted in Bangladesh (20%), India (26%), Iran (18%), Tanzania (21%), Kersa East Ethiopia (28.3%), and Jimma University Specialized Hospital (24.4%) (16, 18, 30–33). This discrepancy may be due to the time gap between studies and recent interventions to prevent low birth weight, which may have contributed to its reduction. Other explanations could include improved access to healthcare facilities, the availability of trained health professionals, prenatal care for pregnant mothers, micronutrient supplementation (iron and folic acid), family planning methods to prevent unwanted and unplanned pregnancies, and antenatal care follow-up.

This study identified that the timing of the first ANC visit, specifically when initiated in the third trimester, was significantly associated with low-birth-weight neonates. Similar to this study, initiating the first ANC visit started in the third trimester was associated with low birth weight in Eastern Nepal (34) and in public hospitals of Addis Ababa (28). This can be explained as early ANC visits, ideally starting in the first trimester, enable healthcare providers to identify, manage, and monitor risk factors affecting fetal growth, such as nutritional deficiencies, maternal infections, or pre-existing medical conditions. Conversely, delaying ANC until the third trimester reduces the opportunity for timely interventions that support fetal growth and development. However, this result is inconsistent with the studies conducted in the University of Gondar and Colombia (25, 35). This difference might be due to variations in healthcare system advancements that can reverse the adverse maternal outcomes irrespective of the time of initiation of ANC follow-up.

The current study also found a significant association between not taking additional nutrition during pregnancy and low birth weight. This finding is consistent with studies conducted in Wolaita Sodo, Addis Ababa, and Dessie town (27, 28, 36). This association may be attributed to the dependence of the fetus on maternal nutrients for optimal growth. During pregnancy, if the mother does not consume additional nutrition beyond her regular diet, there may be insufficient nutrient transfer to the fetus, increasing the likelihood of low birth weight.

The presence of obstetric complications during the current pregnancy was also associated with low-birth-weight neonates, consistent with findings from studies in Indonesia, Gambia, Sudan, Gondar, and Bahir Dar referral hospitals (14, 17, 19, 21). This can be explained as mothers who experience complications such as pre-eclampsia during pregnancy are at a higher risk of delivering low-birth-weight infants compared to those without such complications. Hypertensive disorders and other obstetric complications often lead to placental abruption, which restricts fetal nutrition and blood flow, resulting in low birth weight (22).

This association with obstetric complications was not observed in studies conducted in Iran (18). In some settings, obstetric complications may not show a significant relationship with low birth weight due to factors such as effective antenatal management, access to quality healthcare, and timely interventions. Similarly, a study from Dessie town contradicts this idea (36). The difference might also be attributed to variations in population health profiles and socioeconomic factors, all of which can influence these outcomes.

Gestational age was also significantly associated with the occurrence of low birth weight. This result is consistent with studies conducted in Malaysia, India, Sudan, Northwest Ethiopia, and Mekelle Hospital (21, 31, 37, 38). The association between gestational age and low birth weight is mainly due to the limited time for fetal growth and nutrient accumulation in preterm births. Therefore, lower gestational age inherently limits the time available for fetal development, making it a key predictor of low birth weight.

## 5 Conclusion and recommendation

The prevalence of low birth weight in this study was high. Significant factors associated with low birth weight included initiating the first ANC visit in the third trimester, lack of additional nutrition during pregnancy, the presence of obstetric complications during pregnancy, and a gestational age at birth of less than 37 weeks. In collaboration with stakeholders, the Tigray Regional Health Bureau and Public Health Office should strengthen maternal and child health services, particularly the focused ANC program. Furthermore, healthcare providers are advised to raise awareness about the importance of early ANC visits, emphasize dietary guidance during pregnancy, prioritize prevention and early recognition of complications, and implement risk management strategies. Preventing preterm births remains a key approach to reducing the incidence of low birth weight.

## 6 Strength and limitation of the study

The study incorporates both primary and secondary data collected through face-to-face interviews and chart reviews. However, the findings should be interpreted with certain limitations: the study was conducted at a single site and included only governmental health facilities. Although efforts were made to minimize recall bias, such as reviewing maternal charts for early ultrasound results and early pregnancy human chorionic gonadotropin (HCG) tests to improve the reliability of gestational age estimates based on the last normal menstrual period (LNMP), many mothers were unable to recall their LNMPs.

## Data Availability

The original contributions presented in the study are included in the article/Supplementary Material, further inquiries can be directed to the corresponding author.
